# The Sugarsquare study: protocol of a multicenter randomized controlled trial concerning a web-based patient portal for parents of a child with type 1 diabetes

**DOI:** 10.1186/1471-2431-14-24

**Published:** 2014-01-28

**Authors:** Emiel A Boogerd, Cees Noordam, Chris M Verhaak

**Affiliations:** 1 Department of Medical Psychology, Radboud university medical center, PO Box 9101, 6500 HB, Nijmegen, the Netherlands; 2 Department of Pediatrics, Radboud university medical center, Nijmegen, the Netherlands; 3 Children's Diabetes Center, Nijmegen, the Netherlands

**Keywords:** Diabetes mellitus type 1, Parenting stress, Health communication, Peer support, E-health, Internet

## Abstract

**Background:**

Type 1 diabetes demands a complicated disease self-management by child and parents. The overwhelming task of combining every day parenting tasks with demands of taking care of a child with diabetes can have a profound impact on parents, often resulting in increased parenting stress. Tailored disease information, easy accessible communication with healthcare professionals and peer support are found to support parents to adequately cope with the disease and the disease self-management in everyday life. Internet can help facilitate these important factors in usual pediatric diabetes care. Therefore, we will develop a web-based patient portal in addition to usual pediatric diabetes care and subsequently evaluate its efficacy and feasibility. The web-based patient portal, called Sugarsquare, provides online disease information, and facilitates online parent-professional communication and online peer support. We hypothesize that parenting stress in parents of a child with type 1 diabetes will decrease by using Sugarsquare and that Sugarsquare will be feasible in this population.

**Methods/Design:**

We will test the hypotheses using a multicenter randomized controlled trial. Eligible participants are parents of a child with type 1 diabetes under the age of 13. Parents are excluded when they have no access to the internet at home or limited comprehension of the Dutch language. Participants are recruited offline from seven clinics in the Netherlands. Participants are randomly allocated to an intervention and a control group. The intervention group will receive access to the intervention during the twelve-month study-period; the control group will receive access in the last six months of the study-period. Self-reported parenting stress is the primary outcome in the present study. Data will be gathered at baseline (T0) and at six (T1) and twelve (T2) months following baseline, using online questionnaires. User statistics will be gathered throughout the twelve-month study-period for feasibility.

**Discussion:**

Dependent on its feasibility and efficacy, the intervention will be implemented into usual pediatric diabetes care. Strengths and limitations of the study are discussed.

**Trial registration:**

NTR3643 (Dutch Trial Register)

## Background

Type 1 diabetes (T1D) is a chronic metabolic disorder due to carbohydrate malfunctioning. The incidence of T1D in children is increasing in Europe, with incidence rates expected to raise by 100% in children aged 0 to 5 and by 70% in children aged 0 to 15 in the period from 2005 to 2020 [1]. In 2011, 17.800 new cases of T1D were diagnosed in Europe, increasing the number of children with T1D to 115.700, which makes it the region with the highest rates of children with T1D [2].

T1D comes with a complicated and intrusive treatment regime [3,4]. Parents have to adapt their child’s lifestyle and their own to the demands of the disease, without withholding their child from typical life experiences [5,6]. As such, raising a child with T1D can have great impact on parents’ wellbeing [6,7]. Especially parents of young children with diabetes can show elevated levels of stress, anxiety and depressed mood [8,9], which can lead to an increase in conflicts within the family, and depressed mood and poor self management skills in the child [7,9-11].

Given the impact of the disease and its disease self management, support provided by healthcare professionals is of great importance [4,12,13]. Several aspects in pediatric diabetes care show promising results when it comes to supporting parents. A first important factor is education which, defined as *providing knowledge and skills needed to perform diabetes self-care, manage crises and make lifestyle changes*[12,13], was found to improve quality of disease management and treatment adherence by child and parents [14-17] and to subsequently improve the child’s glycemic control [16,18].

Easy accessible communication with healthcare professionals is a second important factor in diabetes care [19,20]. Tailored and supportive patient-professional communication was found to be associated with improved disease knowledge and quality of disease management of parents, including treatment adherence [20-22]. Literature further points out that parents and patients prefer their healthcare professional to find a balance between exchanging technical information and providing emotional support [20,23].

A third important factor is peer-support, which was found to reduce parenting stress in parents of chronically ill children [24,25] and to reduce the number of parent–child conflicts concerning diabetes in families of a child with T1D [15]. Peer support is also related to better coping in parents of a chronically ill child [25,26]. It is suggested that healthcare professionals should be actively involved in organizing peer contact [15,27], for example by facilitating peer support groups [15] or by appointing mentor-peers [24].

These findings have major implications for healthcare professionals of the diabetes care teams. They are expected to provide tailored disease knowledge, be accessible to patients and facilitate peer support. The internet can be of great assistance to them in facilitating the abovementioned factors [25,28,29]. The role of internet in everyday life has increased significantly during the last decade [27]. Especially pediatric patients who need chronic care and their caregivers are expected to benefit from the potential of the internet, as it can be effectively used for exchange of information and knowledge and lower the threshold for communication with healthcare professionals or peers [29-32]. It is further noteworthy that adolescents with diabetes, parents and healthcare professionals generally support the idea of using internet interventions in pediatric diabetes care [17,33,34].

More research is needed, however, on efficacy and feasibility of internet interventions in pediatric diabetes care and especially concerning interventions that combine multiple aspects of care, such as education, patient-professional communication and peer support [30-32,35].

In the present paper the background, rationale and design of a patient-initiated, multicenter study are described. In the study, a secured web-based patient portal, called Sugarsquare, is developed and evaluated in terms of efficacy and feasibility. The portal integrates the appealing aspect of using internet with providing tailored disease knowledge, easily accessible communication with healthcare professionals of the diabetes team and peer support in a population of parents of a child with T1D.

Hypotheses:

• Usage of Sugarsquare in pediatric diabetes care leads to a decrease in parenting stress in parents of a child with T1D.

• Sugarsquare is feasible in pediatric diabetes care for parents of a child with T1D.

## Methods/Design

### Setting and participants

The present study is conducted in seven clinics for pediatric diabetes care in the Netherlands which, together, deliver care to approximately 750 children with diabetes under the age of 13. Eligible participants are parents of a child with T1D, who receives treatment at one of the seven clinics for diabetes care. Parents are excluded when their child reaches the age of 13 before start of the study. No access to the internet at home and limited comprehension of the Dutch language are also reasons for exclusion.

### Intervention

#### Intervention development and patient participation

The present study was initiated at parents’ explicit request for usage of internet in care. The design and contents of the intervention was partly based on positive results of a comparable intervention implemented in our hospital for couples in IVF treatment [36] and a comparable intervention implemented in our hospital for adolescents with T1D [37]. To match design and contents of the intervention to parents’ preferences, seven focus groups were conducted among parents. Purpose of these focus groups was to map parents’ experiences, needs and wishes concerning their child’s diabetes care. Also, healthcare professionals affiliated to the cooperating diabetes care teams filled out a questionnaire assessing their experiences providing diabetes care and their wishes for fitting the intervention to their workflow.

A test phase, consisting of a series of small pilots, was conducted in the cooperating clinics. Goal was to fine-tune the intervention and to repair bugs. This iterative process helped significantly to fit the intervention to parents’ preferences and to professionals’ workflow. The test phase ended when bugs were repaired and both parents and professionals felt the intervention was ready for use.

#### Intervention

The final version of Sugarsquare consists of a web-based patient portal which provides disease information, easily accessible contact with the diabetes care team and peer support. In accordance to parents’ preferences, the intervention is organized locally. This means that every clinic has its own secured portal, which is only accessible to healthcare professionals of that particular clinic and parents of children treated at that clinic. Sugarsquare is accessible through the internet and consists of three main sections:

Section I: Social

This first section includes online peer support and is accessible to all users (parents and healthcare professionals). Peer support is facilitated through a chat-application, a forum-application and a blog-application. Parents and healthcare professionals are able to communicate in real time by using the chat-application. On the forum-application, healthcare professionals and parents can read and post messages, which are open to all users. Since all users contribute to the social section, it will grow out to have great educational value.

Section II: Personal

This second section applies to individual patients and the information exchanged there can therefore only be accessed by the parents of that particular patient and all healthcare professionals of the clinic. The section consists of an application for overview of treatment goals and an application for easy accessible private contact with the healthcare professionals. The treatment goals are composed during regular consultations with the nurse practitioner and can be accessed online by parents and healthcare professionals at any given moment. The application for easy accessible communication with healthcare professionals is used by parents for discussing the child’s treatment and wellbeing. Discussions are accessible to both parents and all professionals of the diabetes care team. This allows healthcare professionals to fine-tune their advice to previously given advices. This application is only used for non-urgent matters.

Section III: Information

**Figure 1:**
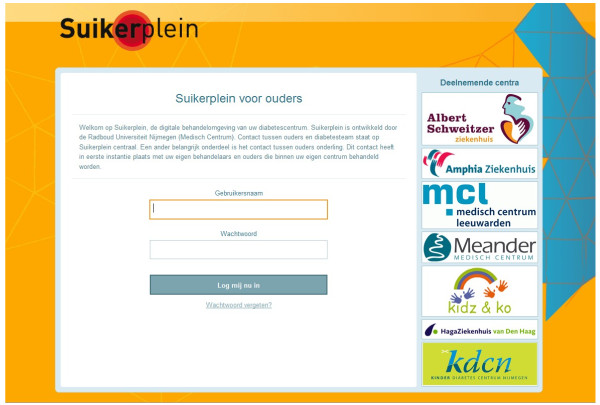
Screenshot of login page of Sugarsquare.

The third section contains disease information, which is presented by means of downloadable documents and web links. Each diabetes care team prepares documents and selects web links. Parents can add web links to Sugarsquare as well, which the diabetes care team can choose to endorse after review. All posted information in this section is available to all users. A screenshot of the login page of Sugarsquare is presented in Figure 1.

#### Access, privacy and security

The main researcher activates the accounts for healthcare professionals and parents. They subsequently receive an invitation e-mail with a request for acceptance of conditions. URL of the intervention, username and password are subsequently provided automatically. When registering, users enter their cell phone number. Sugarsquare is secured by means of a two factor authentication, using a username-password combination and a personalized SMS-code in the login procedure.

#### Diabetes team

All participating clinics provide usual diabetes care by means of a multidisciplinary team, consisting of pediatrician-endocrinologists, nurse practitioners, dieticians and psychologists. The nurse practitioners of the diabetes care team moderate the forum daily, organize weekly chat sessions among participants, fill out the treatment overview during consultations and answer questions of participants posed on Sugarsquare. The pediatricians, dieticians and psychologists are involved upon request of nurse practitioners by writing blogs, answering specified questions of participants or participating in the forum or in chat sessions. Parents who have access to Sugarsquare are requested to use the intervention as much as possible for regular non-urgent communication with the diabetes care team, instead of using conventional tools, such as e-mail or telephone. Besides replacement of communication through telephone- and e-mail in case of non-urgent matters, care as usual will not be altered.

### Study design and procedure

#### Design

A 12-month, multicenter, randomized controlled trial (RCT) is conducted, in which participants are assigned to one of two conditions: 1) an intervention condition and 2) a waiting-list control condition. Participants in the intervention group have access to the intervention during the entire 12-month study period. Participants in the control group are placed on a six-month waiting list. In the remaining six months they will have access to Sugarsquare.

#### Procedures

Eligible, potential participants are approached by their diabetes care team with hard-copy information (information letter, flyer, brochure and application form) about the study. Upon returning the application form, participants are randomized and are subsequently send a questionnaire. When participants have sent back their filled out baseline questionnaire, they are informed about the allocation. Participants who fail to return their baseline questionnaire are contacted by telephone by their diabetes care team, requesting them to send back the filled out questionnaire. After six months following baseline assessment, a second assessment is conducted, also by means of a questionnaire. After having sent back the second questionnaire, participants in the experimental group retain access. Participants in the control group are granted access after having sent back their second questionnaire. After twelve months following baseline assessment, a third assessment will take place, again by means of a questionnaire. Participants who fail to return their second or third questionnaire after request over telephone are considered as drop-out.

All procedures described in this study protocol are approved by the Ethics Committees of Human Experimentation of the Radboud university medical center and of the participating hospitals and are in accordance with the Declaration of Helsinki. Written informed consent will be obtained from all participants.

### Randomization & blinding

Randomization takes place per center and is conducted using envelopes containing red and green cards. For every clinic, there are as many cards as there are participants in the research population. There are as many red cards as there are green cards. Every card is concealed in aluminum foil, so the color will not be visible in any way except when opening the envelope. For every included participant an envelope is picked. When the envelope contains a green card, the participant will be allocated into the experimental group. When the envelope contains a red card, the participant will be allocated into the control group. Randomization is carried out by an independent researcher.

This study is not blinded. Since patient-professional communication is part of the intervention, healthcare professionals know whether a participant is allocated in the experimental group. Parents themselves also know whether they are allocated in the experimental group or the control group. The main researcher is administrator of the intervention and is responsible for enrolment of participants in the intervention and for support during the study period.

### Sample size

We aim to include 240 parents. This number of participants is calculated using a medium effect size (d = .5), an α of .05 (two-tailed test) and a β of .10. The hypothesized effect size is realistic, when considering the paper of Leung et al. ([38]; see also Table 1), in which an effect size of 1.38 was described, using the PSI-SF. To reach adequate power (.90), 180 participants are included in the final analysis [39]. These participants are divided equally into an experimental group (N = 90) and a control group (N = 90). However, in recent studies on randomized controlled trials regarding E-health interventions, an average dropout rate of 25% was found [28]. This means that, when taking drop-out into account and when aiming at 180 participants in the final analyses, at least 240 parents have to be included at the start of the study.

**Table 1:** **Means and standard deviations of the parent-reported measures of the PSI**[38]

**Intervention group**		**Control group**	
**M(SD)**		**M (SD)**	
Pre intervention	Post intervention	Pre intervention	Post intervention
121.60 (17.16)	85.27 (19.91)	112.87 (14.35)	109.08 (14.98)

### Data-collection

Data are collected through self-report questionnaires except for the medical data and user statistics. All questionnaires are sent at baseline, T1 (6 months following baseline) and T2 (12 months following baseline), through the internet. All data are collected using Radquest. Radquest is software used for composing and storing questionnaires using a secured server and was developed by the department of Medical Psychology of the Radboud university medical center. Data derived from patients’ medical files are gathered by nurse practitioners of the diabetes care teams.

### Study outcome measures

#### Background variables

Demographics are gathered through questionnaires on baseline only (see Table 2).

**Table 2:** Background variables used in the Sugarsquare study

**Background variables**	**Measures**
Demographics	• Age and gender of the child
	• Onset and duration of diabetes
	• Pen or pump treatment
	• Age, gender and educational level of the primary parent
	• Social economic status of the parents

#### Primary and secondary outcome measures

Standardized questionnaires are used to gather data on primary and secondary outcome measures.

Parenting stress is the primary study parameter. We aim to assess parenting stress by means of the Dutch version of the Parenting Stress Index- short form (PSI-SF [40]). The reliability and criterion validity of the Dutch PSI-SF are shown to be good [41]. The PSI-SF consists of 25 items which can be answered using a 6-point lykert-scale, ranging from ‘totally agree’ to ‘totally disagree’. An example of an item on the PSI-SF is ‘it is not always easy to accept my child the way he/she is’. The sum score on the PSI-SF can be categorized into normal, subclinical, and clinical based on standardized cut-off scores described in the manual [40].

For an elaborate overview of secondary outcome measures, see Table 3. Most mentioned questionnaires (PEQ-D, DKT, CIDS, DFCS, PedsQL) have been developed and validated especially for research in diabetes care. The general questionnaires (GHQ-12, SDQ, MMAS) have demonstrated good psychometric properties in the general pediatric population.

**Table 3:** Primary and secondary outcome measures used in the Sugarsquare study

**Outcome**	**Measure**
*Primary outcome*	
Parenting stress	Parenting Stress Index- short form (PSI-SF [40])
*Secondary outcomes*	
Parents’ psychosocial wellbeing	General Health Questionnaire (GHQ-12 [42])
Parents’ satisfaction of quality of diabetes care	Patients’ Evaluation of Quality of care- Diabetes (PEQ-D [43])
Parents’ knowledge about diabetes (care)	Diabetes Knowledge Test (DKT [44])
Parents’ treatment adherence	Morisky Medicine Adherence Scale (MMAS [45])
Parents’ confidence in diabetes self-care	Confidence In Diabetes Self-care questionnaire (CIDS [46])
Diabetes related conflicts	Diabetes Family Conflict Scale (DFCS [47])
The impact of diabetes on the family	Pediatric Quality of Life Inventory - family impact scale (PedsQL FIS [48]
The child’s quality of life	Pediatric Quality of Life Inventory - generic scale - parent report (PedsQL generic [49])
The child’s health-related quality of life	Pediatric Quality of Life Inventory - diabetes module - parent report (PedsQL-DM [49])
The child’s psychosocial well-being	Strength and difficulties questionnaire - parent report (SDQ [50])

#### Feasibility

In present literature, a variety of approaches to assess feasibility can be found [37,51]. As to use a more standardized measure, Bowen and colleagues [51] suggest focusing on several areas of feasibility of an intervention: They distinguish between acceptability, demand, implementation, practicability, adaptation, integration, expansion and efficacy. Which of the eight area of focus are assessed depends on the goal of the study and interest of the researchers [37,51]. In this study we concentrate on practicability, acceptability, demand and integration (see Table 4). For assessment of feasibility, individual user data, such as frequency of logins and number of messages posted on the forum, are logged automatically and digitally (see also Table 4).

**Table 4:** Feasibility measures used in the Sugarsquare study

**Outcome**	**Measures**
Practicability (can they use it?)	• Percentage of users who logged in at least once
	• Inventory of difficulties logging in
	• Inventory of downtime (inaccessibility)
Acceptability (do they use it?)	• Percentage of users who logged in at least once and used all applications
	• Duration of usage
Demand (do they continue to use it?)	• Percentage of users who logged in repeatedly
Integration (does it fit with the treatment?)	• Evaluation of international guidelines for diabetes care (ISPAD/IDF/ADA) when using Sugarsquare

These user data can subsequently be associated with potentially reported change over time. This will give insight in efficacy of the separate applications. In addition to actual usage, data on users’ experiences with and evaluation of the separate applications on the intervention are gathered, using a questionnaire on T2.

#### Other outcomes

Information on the child’s glycemic control (HbA1c) and the number of hospital admissions of 24 hours or over in case of keto-acidosis or severe hypoglycemia, throughout the entire study-period are derived from the child’s medical files (see Table 5).

**Table 5:** Other measures used in the Sugarsquare study

**Outcome**	**Measures**
Medical parameters	• HbA1c
	• Hospitals admissions due to glycemic disruptions

### Analyses

#### Descriptive statistics

Demographics of the research sample will be analyzed descriptively. Secondly, differences at baseline between subpopulations and clinics will be assessed by using analysis of variance (ANOVA).

#### Primary analysis

To compare differences between treatment and control group on our primary outcome measure on T0 and T1, analyses of covariance (ANCOVA) will be performed on T1 data, using T0 data as covariates. Effects for clinic differences will be taken into account. A sensitivity analysis will be conducted by means of a multiple imputation analysis (based, among others on HbA1c scores of the population at T1) and an analysis based on a Last Observation Carried Forward (LOCF) imputation.

#### Secondary analysis

Similar analyses are conducted for exploring effects on secondary outcome measures and medical parameters. Data on T2 is regarded as follow-up.

#### Feasibility

For feasibility, user data will be explored, by means of descriptive statistics. Association of user data with individual characteristics on baseline, change on primary and secondary outcome measures and medical parameters will be explored, using Pearson Correlations Coefficients and univariate ANOVA.

## Discussion

This paper describes the protocol for a multicenter randomized controlled trial, by which the efficacy and feasibility of a web-based patient portal will be evaluated, in a population of parents of a child with T1D. The web-based patient portal, called Sugarsquare, integrates the appealing aspect of using internet with providing education, easy accessible contact with the diabetes team, and peer support. We hypothesize that the intervention will decrease parenting stress in parents of a child with T1D and will be feasible in the research population.

Sugarsquare provides patients and healthcare professionals with an innovative and easy-accessible tool. Sugarsquare is expected to support parents in coping with and learning about diabetes through exchange of experiences and ideas with peers and to ease communication between parents and healthcare professionals. It is also expected to be feasible as it contributes to the multidisciplinary character of diabetes care by making all communication between healthcare professionals and parents visible for and accessible to all involved healthcare professionals. Combining those aspects in one intervention is an important strength of our study. A second strength of this study is that it incorporates patient participation in the development of the intervention. By exploring needs and wishes of the users and by extensive piloting of the intervention, we were able to fine-tune the intervention to users’ preferences [17,52]. This will contribute to usability of the intervention and to its generalizability when implementing the intervention in daily care. A third strength of this study is the design of the feasibility assessment using domains proposed by Bowen [51]. This design will enable us to link usage of different sections or applications in the intervention to change in different domains. This design is, on the other hand, also a vulnerability. A design using one arm for every section or application would make it easier to assess the separate contribution of individual sections or applications. However, a web-based patient portal such as Sugarsquare highly depends on the number of users [31]. In the Netherlands, Diabetes care is organized locally, resulting in a great number of diabetes teams with relatively small populations. To get enough users in the intervention group, we could only take on two arms in the present study: 1) an experimental arm and 2) a control arm. Another vulnerability is the chance for drop-out. Studies on internet-delivered interventions often suffer from high drop-out rates, which can significantly interfere with finding potential efficacy of the intervention [28]. To minimize interference, we took a drop-out rate of 25% into account in the sample size calculation. However, we can still be confronted with problems regarding power in the intention-to-treat analysis.

In conclusion, a significant portion of parents of a child with T1D report high levels of parenting stress. Disease education, easy accessible communication with the diabetes care team and peer support help in reducing stress. Although these modes of support are suitable for delivery through the internet, effects of web-based delivery of these aspects in pediatric diabetes care are hardly described in literature. The present study aims to contribute to the knowledge on effects of a web-based patient portal on parenting stress and its feasibility in a population of parents of a child with T1D. Depending on its efficacy and feasibility, the intervention will be attuned in light of results of the study and additionally be implemented in usual pediatric diabetes care.

## Competing interests

The authors declare that they have no competing interests.

## Authors’ contributions

All authors participated in the design of the study. EB drafted the manuscript. CV and KN edited the manuscript. All authors have read and approved the final manuscript.

## Authors’ information

CV, PhD, is a clinical psychologist and section leader of patient care section for the Department of Medical Psychology of the Radboud university medical center. Professor CN MD, PhD, is head of the Department of Pediatrics of the Radboud university medical center and medical director of the Children’s Diabetes Center. EB, MSc, is a PhD-student at the Department of Medical Psychology of the Radboud university medical center.

## Pre-publication history

The pre-publication history for this paper can be accessed here:

http://www.biomedcentral.com/1471-2431/14/24/prepub
